# Application of an *in vitro* neuroinflammation model to evaluate the efficacy of magnesium-lithium alloys

**DOI:** 10.3389/fncel.2024.1485427

**Published:** 2024-10-29

**Authors:** Krathika Bhat, Heike Helmholz, Regine Willumeit-Römer

**Affiliations:** Institute of Metallic Biomaterials, Helmholtz-Zentrum Hereon, Geesthacht, Germany

**Keywords:** neuroinflammation, glial cells, lithium, magnesium, bipolar disorder, *in vitro*

## Abstract

Mg-Li alloys can be promising candidates as bioresorbable Li-releasing implants for bipolar disorder and other neurodegenerative disorders. In order to compare the therapeutic efficacy of conventional Li salts and Li delivered through Mg-Li alloy extracts, we tested an *in vitro* model based on the neuroinflammation hypothesis of mood disorders (peripheral inflammation inducing neuroinflammation) wherein, a coculture of microglia and astrocytes was treated with conditioned medium from pro-inflammatory macrophages. Two alloys, Mg-1.6Li and Mg-9.5Li, were tested in the form of material extracts and well-known outcomes of Li treatment such as GSK3β phosphorylation (indirect flow cytometry) and influence on inflammation-related gene expression (qPCR) were compared against Li salts. This is the first study demonstrating that Li can increase the phosphorylation of GSK3β in glial cells in the presence of excess Mg. Furthermore, Mg-Li alloys were more effective than Li salts in downregulating *IL6* and upregulating the neurotrophin *GDNF*. Mg had no antagonistic effects toward Li-driven downregulation of astrogliosis markers. Overall, the results provide evidence to support further studies employing Mg-Li alloys for neurological applications.

## 1 Introduction

Bipolar disorder (BD) is a chronic disorder that is marked by episodes of mania and depression (Vieta et al., 2018) and the incidence has increased from 79.21 per 100,000 in 1990 to 84.97 per 100,000 people in 2019, affecting more than 1% of the world population (Zhong et al., 2024). Lithium (Li) was one of the first treatments approved for BD and remains competent even today (Vieta et al., 2018). Li has many biological effects like modulation of neurotransmitter systems, anti-inflammatory effects, increased expression of neurotrophins [brain- and glial cell-derived neurotrophic factors (BDNF/GDNF)] and anti-apoptotic factors (Malhi et al., 2013; Ghanaatfar et al., 2023). The inhibition of the enzyme glycogen synthase kinase (GSK3β) by Li is commonly reported to be involved in its mood stabilization effect. Li can inhibit GSK3β directly by competing with the cofactor magnesium (Mg) for its binding site and indirectly through GSK3β phosphorylation by activating the phosphoinositide 3-kinase/protein kinase B (PI3K/Akt) pathway (Rowe et al., 2007; Snitow et al., 2021). GSK3β inhibition and Akt activation can increase the activity of CRE-binding protein [CREB, CRE: cyclic adenosine monophosphate (cAMP) response element] which is responsible for the transcription of neuroprotective factors (Ghanaatfar et al., 2023).

Li treatment is associated with many side effects due to its narrow therapeutic range (0.6–1 mM) (Gitlin, 2016). Implant-based local Li delivery could not only maintain consistent drug levels in the target tissue and reduce side effects (Adepu and Ramakrishna, 2021), but also improve patient adherence to drugs, which is a challenge in patients with mood disorders (Irani et al., 2004). We recently published a study introducing Mg-Li thin film alloys as Li-releasing implants, showing that they are cytocompatible and do not evoke glial cell reactivity *in vitro* (Hanke et al., 2023; Bhat et al., 2024). Mg can be a bioresorbable, functional carrier material in the nervous system (Li et al., 2022) as it plays a role in neurotransmitter balance and Mg deficiency can lead to apoptosis and low-grade neuroinflammation (Maier et al., 2022). The neurological applications of Mg-based materials have been limited to studies with nerve guidance conduits, cerebrovascular stents and temporary electrodes for recording neural activity (Li et al., 2022). In order to support the application of Mg-Li alloys as Li-releasing implants, it is necessary to confirm if Li delivered through Mg-Li alloys have comparable effects to traditionally administered Li.

In order to study the beneficial effects of Mg-Li alloys, a disease-relevant model was necessary. However, *in vitro* models and animal models for BD are limited due the complex polygenic etiology of BD and the lack of specific biomarkers (Quadrato et al., 2016). Use of induced pluripotent stem cells (iPSC) from BD patients is a promising approach but it comes with some challenges such as access to patient material, maintaining the viability of iPSCs and achieving mature cellular traits (Quadrato et al., 2016). A prevailing theory in BD etiology is the neuroinflammation hypothesis. Studies have found increased serum levels of inflammatory markers such as interleukin (IL)-1, IL-6, tumor necrosis factor (TNF)α, soluble receptors of TNF, and IL-6 in BD patients (Benedetti et al., 2020). This is attributed to aberrant inflammatory gene expression from peripheral monocytes which occurs due to genetic abnormalities (Padmos et al., 2008). Chronically high levels of peripheral inflammatory factors could induce neuroinflammation which contributes to the origin and progression of BD (Benedetti et al., 2020), although this is still a debated topic (Sneeboer et al., 2019).

In this study, we used an *in vitro* model based on the neuroinflammation hypothesis of mood disorders to evaluate the therapeutic effects of Li delivered through Mg-Li alloy extracts. In brief, a monocytic cell line was differentiated to macrophages and stimulated to shift toward a pro-inflammatory phenotype. In the brain, glial cells like microglia and astrocytes play a key role in neuroinflammation (Almeida et al., 2020). Hence, a co-culture of microglial and astrocytic cell lines was treated with the conditioned medium from the macrophages and driven toward a neuroinflammatory state. Then, extracts prepared from Mg-Li alloys were added to the glial co-culture and their influence on cellular responses relevant to inflammation and neuroprotection were studied.

## 2 Materials and methods

### 2.1 Preparation of Mg and Mg-Li material extracts

Mg disks (9 mm diameter, 1.5 mm height), Mg-1.6Li and Mg-9.5Li thin films (10 × 10 mm) were used in this study. The fabrication process and material characterization have been published in detail elsewhere (Hanke et al., 2023). The materials were cleaned by submerging (thin films) or sonication (disks) in n-hexane, acetone (20 min each), and pure ethanol (3 min). They were sterilized by submerging in 70% ethanol (20 min) and allowed to dry under sterile conditions. The materials were incubated in Minimum Essential Medium (MEMα, 12571063, Thermo Fisher, Germany) supplemented with 10% fetal bovine serum (FBS, S0615, Merck, Germany) at a ratio of 1 film (~3 mg) per mL for 72 h under cell culture conditions (37°C, 5% CO_2_, 95% relative humidity). The supernatants were sterile filtered (0.2 μm) and kept at 4°C until use. The Mg and Li concentrations in the extracts were determined by inductively coupled plasma with optical emission spectroscopy (SPECTROS-ARCOS, SPECTRO Analytical Instruments, Germany).

### 2.2 LPS treatment of U937-derived macrophages

The U937 (ATCC CRL-1593.2, procured from Deutsche Sammlung von Mikroorganismen und Zellkulturen, Germany) cells were grown until a density of 500,000 cells/mL in RPMI-1640 medium (R0883, Merck, Germany) supplemented with 10% FBS, 2 mM L-glutamine (25030081), 10 mM HEPES (15630080) (both Thermo Fisher, Germany), and 1 mM sodium pyruvate (P5280, Merck, Germany) and differentiated to macrophages (hence called D'U937) by exposing them 10 ng/mL phorbol 12-myristate 13-acetate for 48 h. After differentiation, the medium was replaced with MEMα+10% FBS and the cells were allowed to adapt for 24 h. D'U937 cells were treated with 10 ng/mL bacterial lipopolysaccharide (LPS, *E. coli* O26:B6, L2654, Merck, Germany) together with 5 ng/mL interferon gamma (IFNγ, 11343534, ImmunoTools, Germany) for 24 h. Then, this conditioned medium (henceforth called LPSCM) was sterile filtered and stored at −80°C until use. Similarly, MEMα+10% FBS with LPS+IFNγ (shortly called LPS) and conditioned medium from D'U937 cells exposed to MEMα+10% FBS (D'U937CM) were prepared to differentiate the effects of factors secreted by LPS-stimulated D'U937 cells.

The gene expression changes in cytokines and chemokines in LPS-stimulated D'U937cells were identified using a qPCR array (330231 PAHS-150ZD-2, Qiagen, Germany). For this purpose, RNA was extracted using the Quick-RNA™ Microprep kit (R1051, Zymo Research, Germany) and cDNA synthesis was performed using the RevertAid RT Kit (K1691, Thermo Fisher, Germany) as per the manufacturer's protocols. The qPCR reaction was carried out using PerfeCTa SYBR^®^ Green FastMix (95072, Quantabio, VWR, Germany) with cDNA input corresponding to 12.5 ng total RNA per well and CFX Opus 96 PCR system (Bio-Rad, Germany). Cycle threshold (Ct) values were determined from the software output (CFX Maestro, Bio-Rad, Germany). The fold changes in gene expression were calculated using the ΔΔCt method. One array plate was used for unstimulated D'U937 cells and one for LPS-treated cells.

TNFα, IL-1β, and IL-6 in the conditioned media were measured by ELISA using commercially available kits (TNFα: DY210 and IL-1β: DY201, R&D systems, IL-6: 31670069, ImmunoTools, Germany) and ancillary reagents (DY008, R&D systems, Germany). The absorbance was measured at 450 nm and a reference wavelength of 570 nm (Victor^3^V multilabel plate reader, PerkinElmer, Germany).

### 2.3 Influence of U937-derived conditioned medium and material extracts on glial cell coculture

The human microglial cell line HMC3 (kindly provided by Prof. Kirsten Hattermann, University of Kiel, Germany) and SV-40 immortalized astrocytes IM-HA (P10251-IM, Innoprot, Spain) were grown in Dulbecco's modified Eagle's medium (31966047, Thermo Fisher, Germany) with 10% FBS and switched to MEMα+10% FBS for the experiments. For the indirect coculture, HMC3 cells were seeded in inserts (1 μm pore size, PET, Sarstedt, Germany) at 10,000 cells/cm^2^ density and IM-HA cells in the well at 20,000 cells/cm^2^. Cells were seeded in a 12-well plate (WP) for PCR (0.5 mL in insert, 1.5 mL in well) and 6WP for GSK3β phosphorylation analysis (1 mL in insert, 2 mL in well).

After 24 h, the conditioned media were added after 1:1 dilution with fresh medium. After 72 h, the material extracts were added along with LPSCM such that the final concentration of Mg in the wells was 10 mM, the Li concentration corresponded to the alloy (Mg-1.6Li: 0.8 mM, Mg-9.5Li: 4.2 mM) and LPSCM was 50% of the final volume. In addition, wells containing LiCl salts at the same Li concentrations as alloy extracts were included. The medium was refreshed every alternate day and the sampling was carried out after 168 h exposure to material extracts+LPSCM.

#### 2.3.1 Analysis of relative phosphorylation of GSK3β

The relative phosphorylation level of GSK3β (P_rel_) was measured using an indirect flow cytometry protocol. The inserts were moved to a different plate, the cells were washed with pre-warmed PBS and detached using 1 mL of pre-warmed Accutase^®^ (A6964, Merck, Germany) (5 min). The cell suspension was pipetted into 1 mL of 4% paraformaldehyde for fixation (room temperature, 15 min). The cells were washed twice with PBS (400 g, 5 min), 1 mL of ice-cold 90% methanol was added drop-wise and the cells stored at −20°C until staining.

The cells were washed twice with PBS+1% BSA and 50,000 cells were transferred to each well (5 wells/sample) in a round bottom 96WP. The plate was centrifuged (400 g, 5 min) and after removing the supernatant, 100 μL of staining buffer (PBS+1% BSA) containing only Alexa Fluor^®^ 488-conjugated anti-GSK3β antibody (0.5 μg/mL, tGSK, IC2506G, R&D systems, Germany) or along with anti-phospho-GSK3β (Ser9) antibody (1 μg/mL, pGSK, 5558S, Cell Signaling Technology, Netherlands) was added (for each sample: 2 wells each for tGSK and tGSK+pGSK, 1 well without antibodies). The plate was incubated in the dark for 16 h at 4°C (125 rpm shaking). The plate was washed twice and 100 μL of staining buffer containing Texas Red-conjugated secondary antibody (4 μg/mL, 111-075-003, Jackson Immunoresearch, United Kingdom) was added. The plate was incubated in the dark for 1 h at room temperature (125 rpm shaking). The plate was washed thrice, the samples were passed through a cell strainer (70 μm) and analyzed by flow cytometry (S3e Cell Sorter, Bio-Rad, Germany). After gating for cells using FSC-A vs. SSC-A, tGSK fluorescence was detected using the 488 nm laser and 525/30 nm emission filter. For tGSK-positive population, the secondary antibody fluorescence intensity (I_FL3_) was detected using the 561 nm laser and 615/25 nm emission filter. P_rel_ was calculated using I_FL3_ as follows:


$$\begin{array}{l} P_{rel}=\frac{I_{FL3,pGSK+tGSK}}{I_{FL3,tGSK}} \end{array}$$

I_FL3, tGSK_: wells with tGSK (background); I_FL3, pGSK+tGSK_: wells with tGSK+pGSK.

#### 2.3.2 Gene expression analysis

The protocol was similar to Section 2.2. The inserts were separated from the wells and RNA was extracted separately from the cells for cDNA synthesis. Taqman assays were used for qPCR [*GAPDH*: Hs02758991_g1, *SDHA*: Hs00188166_m1 (two housekeeping genes), *IL1B*: Hs00174097_m1, *IL6*: Hs00174131_m1, *GDNF*: Hs00181185_m1, *FN1*: Hs00277509_m1, *TNC*: Hs01115665_m1] with HOT FIREPol^®^ Probe Universal qPCR Mix (081700001, Solis BioDyne, Estonia).

### 2.4 Statistical analyses

Statistical analyses of the data were performed on GraphPad Prism 9.5.0. Data from 3 independent experiments with 2 replicates per run were considered. Outliers were excluded by Grubb's test and significant differences were identified by 1-way ANOVA and Tukey's *post hoc* test.

## 3 Results

### 3.1 Gene expression and cytokine release in LPS-treated D'U937 cells

**Figure 1:**
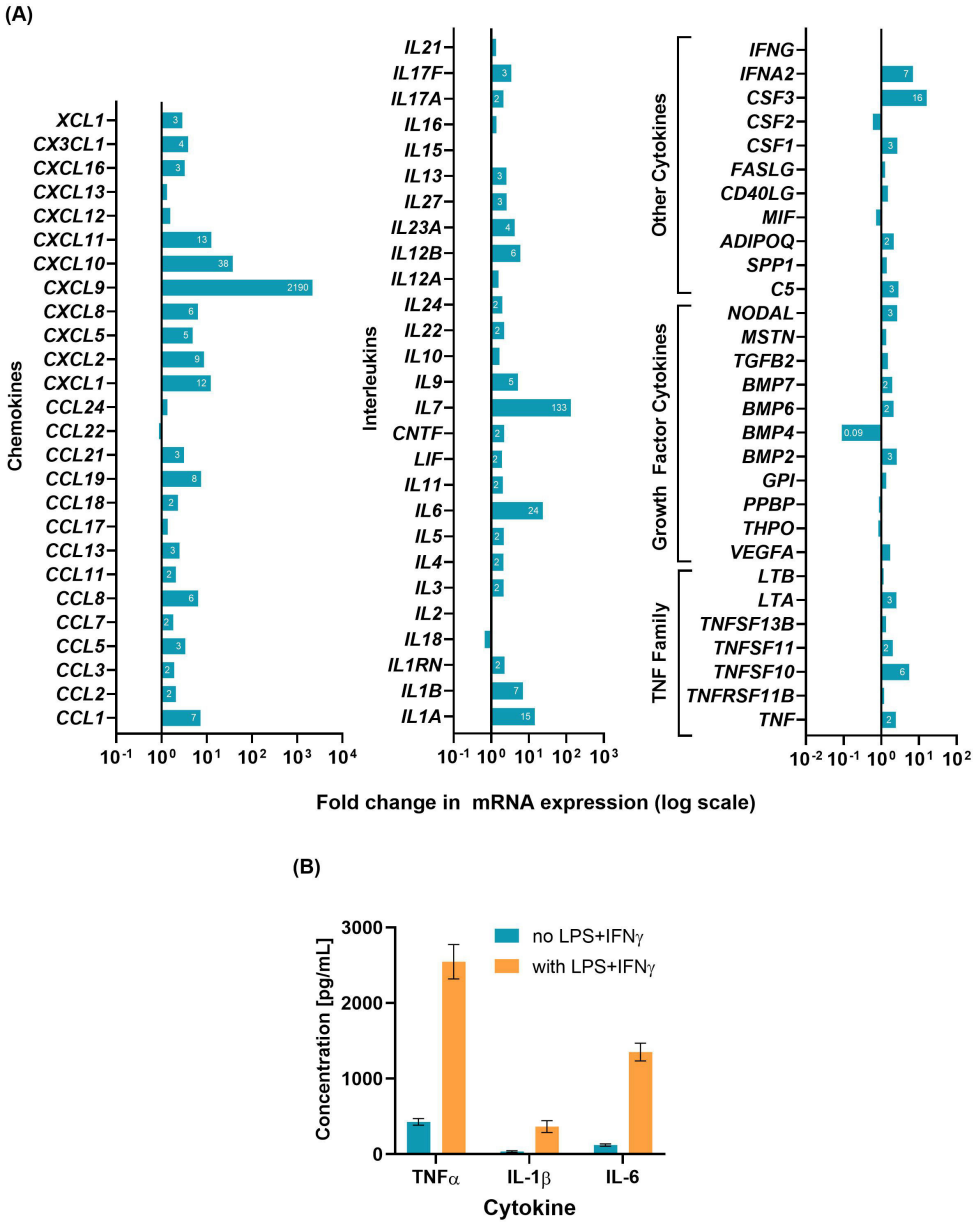
Characterization of D'U937 response to LPS+IFNγ stimulation. U937 monocytes were differentiated to macrophages and treated with 10 ng/mL LPS and 5 ng/mL IFNγ for 24 h. **(A)** Gene expression changes analyzed by a qPCR array. One array plate was used for the untreated cells and one plate for the LPS-stimulated cells. **(B)** Cytokine concentrations in the conditioned media measured by ELISA. Graph shows mean ± SD for results from assays (2 technical replicates per sample per assay) performed on three different aliquots of the conditioned media.

The gene expression changes in D'U937 cells treated with LPS+IFNγ are shown in Figure 1A. The addition of LPS+IFNγ increased the gene expression of several cytokines and chemokines, notably *IL6, IL7, CXCL9*, and *CXCL10*. The cytokine levels measured by ELISA (Figure 1B) revealed increased levels of TNFα, IL-1β, and IL-6 in LPSCM compared to D'U937CM.

### 3.2 Gene expression in glial co-culture due to LPSCM

**Figure 2:**
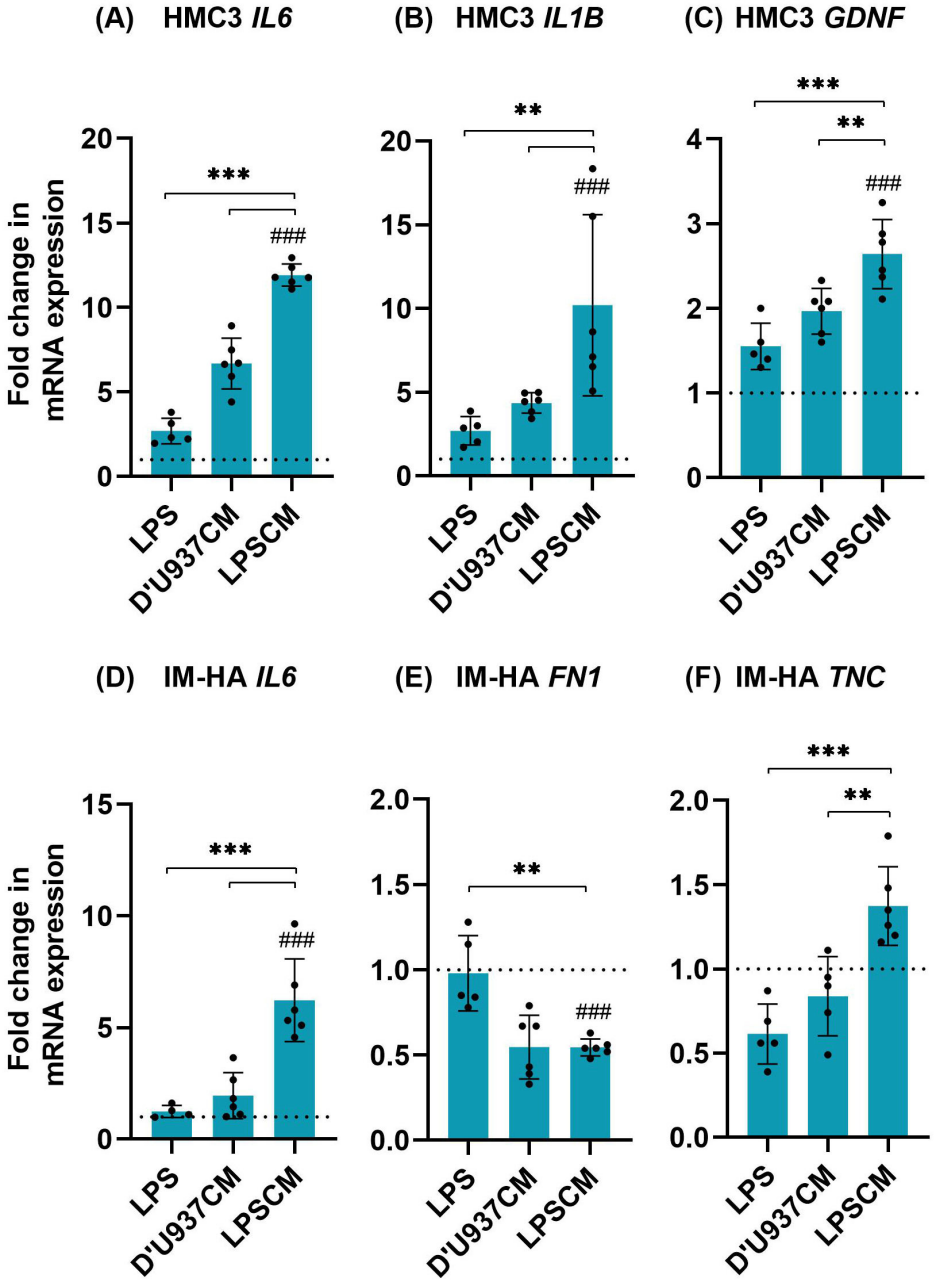
Changes in the expression of HMC3 and IM-HA genes in coculture treated with LPSCM. **(A)** HMC3 *IL6*. **(B)** HMC3 *IL1B*. **(C)** HMC3 *GDNF*. **(D)** IM-HA *IL6*. **(E)** IM-HA *FN1*. **(F)** IM-HA *TNC*. Cells were treated with LPSCM for 10 days and analyzed by qPCR. Graphs show mean ± SD, *n* = 4–6 (*N* = 3). Statistically significant differences were identified by 1-way ANOVA and Tukey's *post hoc* test [F-values: **(A)**: 152.8, **(B)**: 13.07, **(C)**: 33.58, **(D)**: 27.90, **(E)**: 16.25, **(F)**: 9.48]. Symbols: differences to the control, between conditions, *p* < 0.05 (**), *p* < 0.001 (***,###). The dotted line indicates the control value.

To understand the effect of LPSCM on glial cells, untreated coculture (negative control), co-cultures treated with LPS and D'U937CM were compared as shown in Figure 2. Co-cultures exposed to LPSCM showed significantly higher levels of *IL1B* (*p* < 0.01), *IL6* (*p* < 0.001), and *GDNF* (*p* < 0.01) in microglia and *IL6* and *TNC* in astrocytes (*p* < 0.001). *FN1* was significantly downregulated (*p* < 0.01).

### 3.3 Relative GSK3β phosphorylation in glial coculture after treatment with Mg-Li extracts

**Figure 3:**
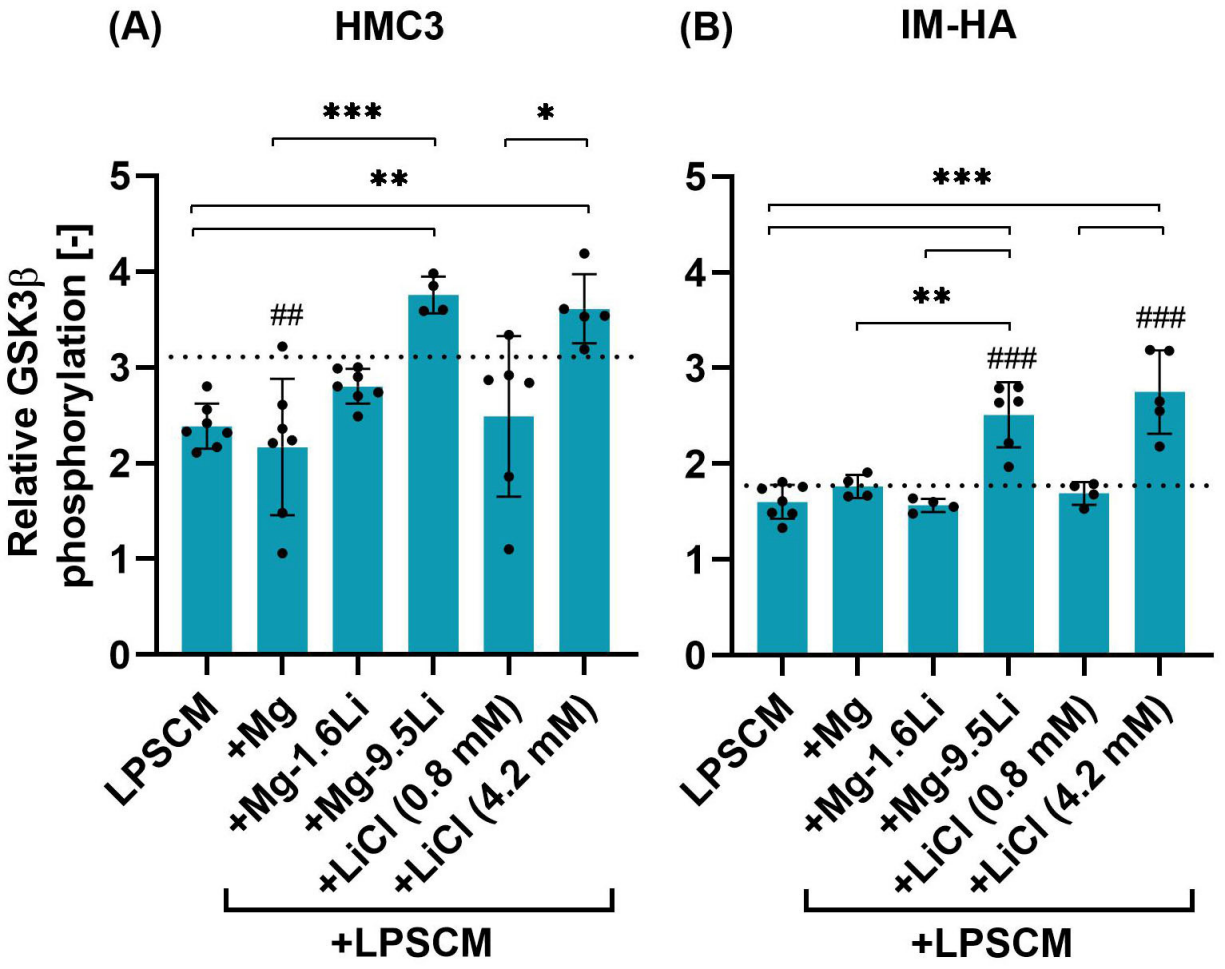
Changes in the relative phosphorylation of GSK3β in HMC3 and IM-HA cells treated with LPSCM and Mg/Mg-Li extracts. **(A)** HMC3. **(B)** IM-HA. Cells were treated with LPSCM for 10 days, of which they were also treated with Mg-Li extracts for 7 days. Graphs show mean ± SD, *n* = 4–6 (*N* = 3). Statistically significant differences were identified by 1-way ANOVA and Tukey's *post hoc* test [F-values: **(A)**: 7.037, **(B)**: 18.08]. Symbols: differences to the control (#), between conditions (*), *p* < 0.05 (*), *p* < 0.01 (**,##), *p* < 0.001 (***,###). The dotted line indicates the control value.

The changes in GSK3β phosphorylation in the co-culture due to LPSCM and Mg-Li extracts are shown in Figure 3. In case of HMC3 (Figure 3A), P_rel_ in the negative control was ~3.1 and addition of LPSCM reduced P_rel_, but not statistically significant. The presence of Mg-9.5Li extract increased P_rel_ significantly compared to cells treated with LPSCM (*p* < 0.01) and LPSCM+Mg extract (*p* < 0.001). The cells treated with 4.2 mM LiCl led to similar results as with Mg-9.5Li extract. Addition of Mg extract, Mg-1.6Li extract and 0.8 mM LiCl did not affect P_rel_ significantly.

In IM-HA cells, the changes in P_rel_ were similar (Figure 3B). A key difference was that the P_rel_ in the negative control was lower and exposure to LPSCM did not change it. Compared to the negative control and LPSCM-treated cells, addition of Mg-9.5Li extract and 4.2 mM LiCl significantly increased P_rel_ (*p* < 0.001). The fluorescence intensity data from flow cytometry measurements are provided in the Supplementary material.

### 3.4 Gene expression changes in glial co-culture due to Mg-Li extracts

**Figure 4:**
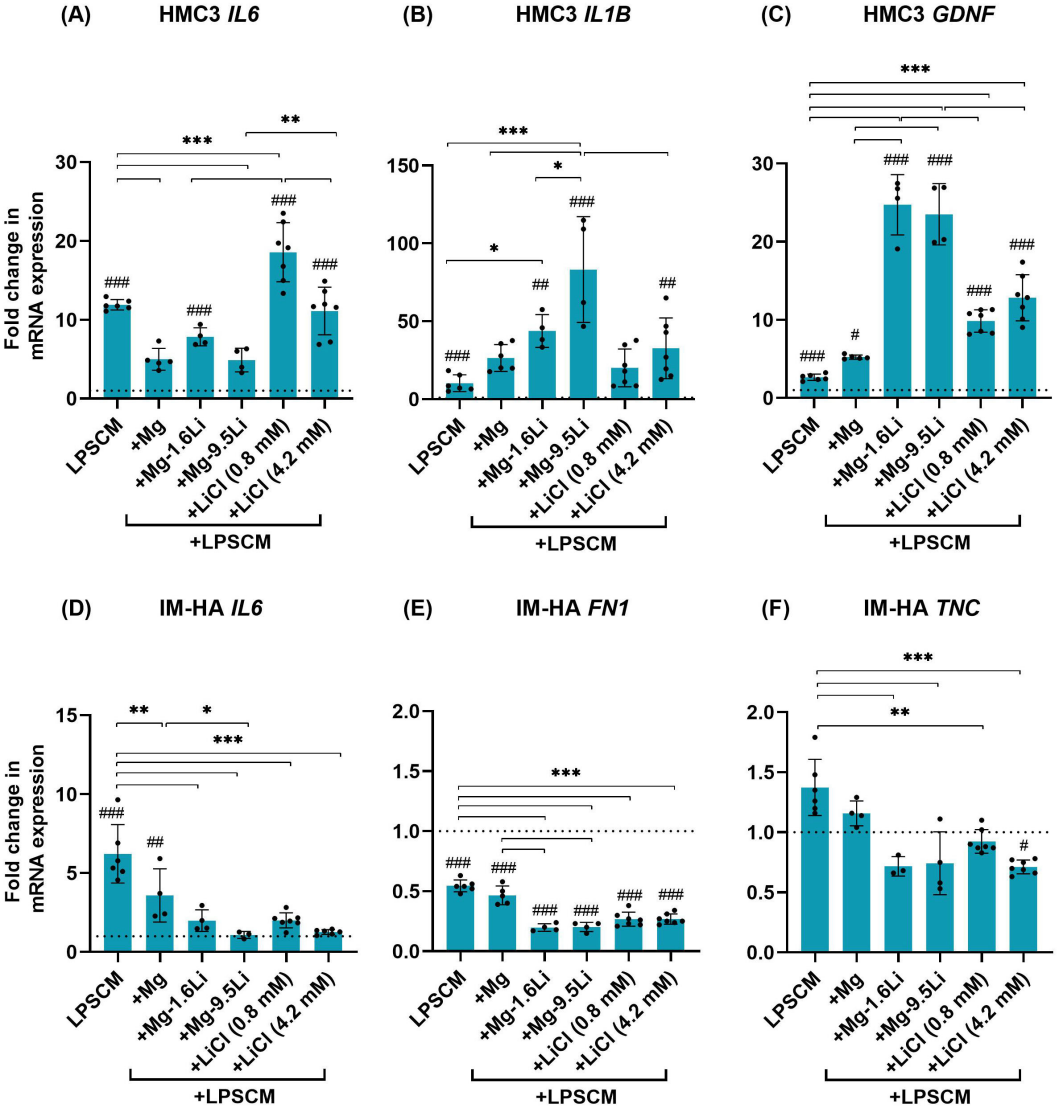
Changes in the expression of HMC3 and IM-HA genes in coculture treated with LPSCM and Mg/Mg-Li extracts. **(A)** HMC3 IL6. **(B)** HMC3 IL1B. **(C)** HMC3 GDNF. **(D)** IM-HA IL6. **(E)** IM-HA FN1. **(F)** IM HA TNC. Cells were treated with LPSCM for 10 days, of which they were treated with extracts for 7 days. Graphs show mean ± SD, *n* = 4–6 (*N* = 3). Statistically significant differences were identified by 1-way ANOVA and *post hoc* test [F-values: **(A)**: 89.84, **(B)**: 15.75, **(C)**: 93.50, **(D)**: 17.52, **(E)**: 90.86, **(F)**: 9.29]. Symbols: differences to the control (#), between conditions (*), *p* < 0.05 (*,#), *p* < 0.01 (**,##), *p* < 0.001 (***,###). The dotted line indicates the control value.

The addition of Mg/Mg-Li extracts to LPSCM-treated HMC3 microglia downregulated *IL6* (Figure 4A, *p* < 0.001), but the influence of Mg-1.6Li extract was statistically insignificant. The influence of both Mg-Li extracts was significantly different compared to Li salts (*p* < 0.01). *IL6* levels due to the addition of 0.8 mM LiCl were higher than with LPSCM alone (*p* < 0.001). Contrastingly, the addition of Mg-Li extracts (Figure 4B) increased *IL1B* levels further (Mg-1.6Li, *p* < 0.05; Mg-9.5Li, *p* < 0.001). The *IL1B* level after LPSCM+Mg-9.5Li treatment was significantly higher than with LPSCM+Mg (*p* < 0.001), LPSCM+Mg-1.6Li (*p* < 0.05), and LPSCM+4.2 mM LiCl (*p* < 0.001). Although *IL1B* levels after exposure to Mg extract and Li salts were also higher compared to LPSCM alone, they did not differ with statistical significance. All Li-containing conditions increased the expression of microglial *GDNF* (*p* < 0.001) compared to LPSCM and LPSCM+Mg conditions (Figure 4C). Moreover, both Mg-Li extracts increased *GDNF* levels significantly higher than the corresponding Li salts (*p* < 0.001).

In IM-HA cells, *IL6* upregulation by LPSCM was significantly downregulated by all extracts and salts (Figure 4D, *p* < 0.001). Compared to Mg extract, Mg-9.5Li extract led to significantly lower levels of *IL6* (*p* < 0.05), but there were no significant differences between the two Mg-Li extracts or the two Li salt concentrations. Astrogliosis markers *FN1* and *TNC* (Figures 4E, F) were downregulated by the addition of Li-containing conditions (*p* < 0.001) but not Mg extract. However, there was no clear effect of Li concentration.

## 4 Discussion

### 4.1 Suitability of the *in vitro* model

In this study, an *in vitro* model for peripheral inflammation inducing neuroinflammation was applied to study the beneficial effects of Mg-Li alloy extracts. Conditioned medium from monocyte cell line-derived macrophages treated with LPS+IFNγ (LPSCM) was used to represent peripheral inflammation. The pro-inflammatory factors in LPSCM caused the glial cells to shift to an inflammatory state, thereby simulating neuroinflammation induced by peripheral factors. Then, Mg/Mg-Li extracts were added and changes in GSK3β phosphorylation and gene expression were analyzed to evaluate their therapeutic effect.

While using patient-derived material such as monocytes and iPSC-derived brain cells would greatly improve the relevance of a BD neuroinflammation model, patient material is not always easy to access. Hence, we tested an alternate approach wherein, U937 monocyte-derived macrophages (D'U937) were treated with a low concentration of LPS to produce a cocktail of pro-inflammatory factors. Since the serum cytokine levels reported in BD patients vary from study to study (Munkholm et al., 2013; Goldsmith et al., 2016) and the findings are often limited to the factors targeted, we used conditioned medium from D'U937 cells (LPSCM) as it could encompass more biological complexity compared to the addition of selected recombinant cytokines. Moreover, since all cell types involved in the model were cell lines and the compounds used for the differentiation/stimulation of U937 cells are widely available, the model should provide good reproducibility.

Padmos et al. (2008) compared monocytes from BD patients to healthy controls and identified several genes to be aberrantly expressed using whole genome gene expression profiling. Among them, *IL1B, IL6, TNF, CCL2, CCL7*, and *CXCL2* were common to the genes targeted in the microarray in this study. In LPS-stimulated D'U937 cells, these genes were only moderately upregulated in comparison to *CXCL9, CXCL10*, and *IL7* which were upregulated several magnitude levels higher. However, the fold changes in mRNA levels of *IL1B, IL6, TNF*, and *CXCL2* were comparable in magnitude and proportion to those reported by Padmos et al. (2008). The most consistently reported cytokines, TNFα, IL-1β, and IL-6, were verified by ELISA to be present in LPSCM at the protein level. The limitation of using a complex, partially characterized stimulant like LPSCM is that factors which may not be relevant to mood disorders, such as LPS, higher levels of chemokines like CXCL9 and CXCL10, could have also influenced the glial cells significantly. Moreover, in the body, the blood-brain barrier controls the crossover of cytokines into the brain environment (Zhao et al., 2022) and this effect was not incorporated in the present *in vitro* model. The model could be improved by separating LPSCM from the glial cells using a basic model of the blood-brain barrier e.g., layers of astrocytes and brain endothelial cells separated by an insert membrane.

LPSCM resulted in clear differences in pro-inflammatory gene expression (*IL1B* and *IL6*) in the glial co-culture, leading to significantly higher levels compared to other conditioned media. *GDNF* upregulation is observed under pro-inflammatory conditions as a protective, anti-inflammatory response (Kotliarova and Sidorova, 2021). Accordingly, a mild upregulation in *GDNF* was observed in LPSCM-treated microglia. Fibronectin (*FN1*) and tenascin C (*TNC*), which are common markers of reactive astrogliosis observed under inflammatory conditions, can also act as toll-like receptor ligands and influence microglia-astrocyte crosstalk (Wiemann et al., 2019). In LPSCM-treated astrocytes, *TNC* was upregulated but contrary to expectations, *FN1* was downregulated. Overall, these results support that physiologically-relevant factors secreted by D'U937 cells contributed to inducing the neuroinflammatory state.

### 4.2 Influence of Mg-Li extracts on GSK3β phosphorylation in glial cells

After exposing the glial coculture to LPSCM to create an inflammatory state, Mg/Mg-Li material extracts were added since our intention is to introduce Mg-Li materials as Li-releasing brain implants in BD patients or for other neurological disorders. Since the inhibition of the enzyme GSK3β is widely considered to be one of the mechanisms of action of Li (Snitow et al., 2021), the phosphorylation status of this enzyme was targeted in glial cells treated with Mg-Li extracts. Li-mediated increase in PI3K/Akt activity is one way by which the inhibitory phosphorylation of GSK3β is increased (Snitow et al., 2021). Interestingly, Mg is also known to increase PI3K/Akt signaling (Yamanaka et al., 2019), and few studies have reported Mg-driven increase in GSK3β phosphorylation as well (Xu et al., 2014; Wang et al., 2018). In this study, both Mg-9.5Li extract containing 4.2 mM Li and LiCl salt of the same Li concentration increased GSK3β phosphorylation to comparable levels in both HMC3 and IM-HA cells. These results demonstrate for the first time that Li can increase inhibitory GSK3β phosphorylation in glial cells, even in the presence of excess Mg and material degradation products. Considering the importance of Li-mediated GSK3β inhibition in its therapeutic effect, this observation is an important outcome with regard to the applicability of Mg-Li materials as Li-delivery implants.

Mg-1.6Li extract which contained serum therapeutic levels of Li (0.8 mM Li) did not have a significant influence on the GSK3β phosphorylation in this study. However, since LiCl at the same concentration also did not significantly increase GSK3β phosphorylation, it is likely that the degradation products in the extract were not the cause. It is possible that the analytical method used here was not sensitive enough to detect the influence of Mg extract and lower Li concentrations. Another important aspect to be considered is that only the phosphorylation-mediated influence of Mg-Li extracts on GSK3β activity was measured. The direct effect on Li^+^ on GSK3β activity through competition with Mg^2+^ (cofactor) binding site was not accounted for and it is unknown if this occurs under conditions of excess Mg^2+^ as found in the extract. To better understand this, *in silico* approaches or an assay to measure total GSK3β activity in living cells treated with Mg-Li extracts would be useful (Zotter et al., 2017).

### 4.3 Influence of Mg-Li extracts on inflammation-related gene expression in glial cells

The gene expression analysis revealed that the material extracts drastically upregulated *IL1B* and a Li dependent increase in fold change was observed. On the other hand, the Li salts did not increase *IL1B* levels to the same extent, suggesting that it was not related only to the Li concentration. Although some studies have reported a Li-induced upregulation in *IL1B* with BD patient-derived material (Knijff et al., 2007; Osete et al., 2023), Mg and Li are commonly reported to suppress pro-inflammatory *IL1B* expression, often in a nuclear factor-κB (NF-κB) dependent manner (Gao et al., 2013; Khan et al., 2017). Since LPSCM was a complex inflammatory stimulant, it could have activated other pathways e.g., extracellular signal regulated kinase (ERK)-dependent pathways (Lu et al., 2007; Chen et al., 2021) which both Mg and Li are known to activate further (Liu et al., 2017; Kong et al., 2019). Therefore, future studies verifying a synergistic effect of Mg and Li on inflammation-driven ERK activation are necessary.

In contrast to *IL1B*, Mg/Mg-Li extracts downregulated *IL6* in HMC3 and the material extracts were more effective than Li salts, suggesting that a mechanism common to Mg and Li might be at play. This observation is intriguing since both *IL1B* and *IL6* have many regulatory transcription factors in common (Khalaf et al., 2010; Adamik et al., 2013). Depending on the cell type and stimulus, studies have found that the inhibition of NF-κB alone can drastically reduce *IL6* expression (Dendorfer et al., 1994; Khalaf et al., 2010). Since both Mg and Li can inhibit NF-κB mediated transcription, their additive effect might have made the difference in case of the material extracts.

In IM-HA cells, Mg-9.5Li extract downregulated *IL6* more than the Mg extract, almost down to the level of the negative control, suggesting a Li mediated effect. This can be linked to the increased inhibitory phosphorylation of GSK3β, since GSK3β inhibition can also reduce IL-6 production through CREB and Janus kinase (JAK)/signal transducer and activator of transcription 3 (STAT3)-mediated pathways (Beurel and Jope, 2009). IL-6 imbalance is often implicated in BD and Vadodaria et al. (2021) showed that BD iPSC-derived astrocytes produced aberrantly high amounts of IL-6 compared to healthy astrocytes. Knijff et al. (2007) reported that monocytes in BD patients have an imbalance in the expression of IL-1β and IL-6, leading to lower levels of IL-1β and higher IL-6 and that Li treatment restores the balance. Therefore, the downregulation of *IL6* by Mg-Li extracts could have important therapeutic consequences in BD.

Another biological outcome linked to the therapeutic effect of Li is the increased expression of neurotrophic factors like BDNF and GDNF (Ghanaatfar et al., 2023; Tsybko et al., 2017). Mg-Li extracts upregulated microglial *GDNF* significantly higher than Li salts, but Mg extracts did not have a major influence on *GDNF*. On the one hand, this could be a beneficial effect of Li that was mediated through increased CREB levels (Tsybko et al., 2017). On the other hand, it could be related to the upregulation in *IL1B* observed under these conditions (Tanabe et al., 2009).

A genomic analysis of Li-treated astrocytes by Rivera et al. revealed that the genes most influenced by Li were involved in astrogliosis and extracellular matrix proteins (Rivera and Butt, 2019). Accordingly, in this study, both Mg-Li extracts and Li salts downregulated the astrogliosis markers *FN1* and *TNC*, and the presence of Mg or material degradation products did not affect this. Astrogliosis markers are closely linked to the activation of STAT3 involving GSK3β (O'Callaghan et al., 2014) and the inhibition of GSK3β by Li might have played a role in the observed attenuation of *TNC* and *FN1*.

## 5 Conclusion

An *in vitro* neuroinflammation model for mood disorders without patient-derived biological material was tested, showing that inflammatory factors from peripheral monocytes can induce neuroinflammation in a glial co-culture differently than conventionally used stimulants like LPS. Li delivered through Mg-Li materials had several comparable therapeutic effects to Li salts in glial cells and the presence of Mg did not antagonize this, the exception being the increased gene expression of IL-1β due to Mg-Li extracts. With improved understanding, Mg has the potential to be a functional and supportive carrier material for Li delivery in the form of Mg-Li alloy-based implants. Since the dysregulation of GSK3β and neuroinflammation are also involved in other neurodegenerative disorders and injury, these results can support the application of Mg-Li biomaterials in other disorders as well.

## Data Availability

The datasets presented in this study can be found in online repositories. The names of the repository/repositories and accession number(s) can be found in the article/Supplementary material, with qPCR array data available in the public repository Gene Express Omnibus (NCBI), series GSE275490.
